# UiO-66-NH_2_/GO Composite: Synthesis, Characterization and CO_2_ Adsorption Performance

**DOI:** 10.3390/ma11040589

**Published:** 2018-04-11

**Authors:** Yan Cao, Hongmei Zhang, Fujiao Song, Tao Huang, Jiayu Ji, Qin Zhong, Wei Chu, Qi Xu

**Affiliations:** 1 School of Environmental Science and Engineering, Yancheng Institute of Technology, Yancheng 224000, China; hjxyzhm@ycit.cn (H.Z.); song_fjiao2006@126.com (F.S.); huangtaoasd@126.com (T.H.); wm12142005@163.com (J.J.); xqsteve@ycit.cn (Q.X.); 2 School of Chemical Engineering, Nanjing University of Science and Technology, Nanjing 210094, China; zhongqin304@163.com; 3 Department of Chemical Engineering, Sichuan University, Chengdu 610065, China; chuwei1965@foxmail.com

**Keywords:** graphene oxide (GO), metal-organic framework (MOF), UiO-66-NH_2_, carbon dioxide adsorption

## Abstract

In this work, a new composite materials of graphene oxide (GO)-incorporated metal-organic framework (MOF)(UiO-66-NH_2_/GO) were in-situ synthesized, and were found to exhibit enhanced high performances for CO_2_ capture. X-ray diffraction (XRD), scanning electron microscope (SEM), N_2_ physical adsorption, and thermogravimetric analysis (TGA) were applied to investigate the crystalline structure, pore structure, thermal stability, and the exterior morphology of the composite. We aimed to investigate the influence of the introduction of GO on the stability of the crystal skeleton and pore structure. Water, acid, and alkali resistances were tested for physical and chemical properties of the new composites. CO_2_ adsorption isotherms of UiO-66, UiO-66-NH_2_, UiO-66/GO, and UiO-66-NH_2_/GO were measured at 273 K, 298 K, and 318 K. The composite UiO-66-NH_2_/GO exhibited better optimized CO_2_ uptake of 6.41 mmol/g at 273 K, which was 5.1% higher than that of UiO-66/GO (6.10 mmol/g). CO_2_ adsorption heat and CO_2_/N_2_ selectivity were then calculated to further evaluate the CO_2_ adsorption performance. The results indicated that UiO-66-NH_2_/GO composites have a potential application in CO_2_ capture technologies to alleviate the increase in temperature of the earth’s atmosphere.

## 1. Introduction

Metal-organic frameworks (MOFs) are formed by the coordination between metal clusters and organic linkers. Due to their large capacity for the adsorption of gases and structural and chemical tenability, they have gained much attention to the potential applications of CO_2_ capture [1,2]. The UiO-66 sample with good thermal stability (>500 °C) and water resistance shows some improved CO_2_ adsorption performance [3,4,5]. Moreover, when compared with the performance of industrial adsorbent (zeolite 13x, for example), the adsorption capacity of UiO-66 demonstrates an improved value [1,6]. In order to enrich this type MOFs, some functional groups were introduced to the skeleton to synthesize the functional series UiO-66 type of MOFs, which could maintain the topology of the original constant [7,8,9,10]. However, after functionalization, although the series UiO-66 type of MOFs still displayed the same skeletal structure, the thermal stability, chemical stability, etc., exhibited degradation to some extent. Kandiah et al. [11,12] compared the thermal stability and the chemical stability of three kinds of UiO-66 types of MOFs (UiO-66-NH_2_, UiO-66-NO_2_, and UiO-66-Br), and the results showed that, for UiO-66-NH_2_ and UiO-66-NO_2_, the thermal stability fell at 350–400 °C, which was due to the ligand decomposition. Moreover, UiO-66-NH_2_, UiO-66-NO_2_, and UiO-66-Br prepared in water and HCl solution with pH = 1 displayed the stability of the structure, while UiO-66-NH_2_ and UiO-66-Br that were prepared in the NaOH solution with pH = 14 exhibited the collapsed structure partially or completely at the above experimental condition. Yang et al. [13,14] reported that, when compared to UiO-66, the CO_2_ adsorption capacity of UiO-66-NH_2_ increased, which was due to the existence of amino. However, the stability of UiO-66-NH_2_ was inferior to that of UiO-66. Therefore, it is necessary to find a new kind of UiO-66 type of MOFs, which can display outstanding performance on thermal stability, chemical stability, and gas adsorption. 

Simultaneously, as reported in the literature, the introduction of NH_2_ could improve the CO_2_/N_2_ selectivity in the coal-fired flue gas [15,16]. MOFs have plenty of porosities, but the large pore space in MOFs was not in favor of gas molecules storage. GO has a layered structure and its surface has some groups (–OH, C–O–C, –C=O). The incorporation of GO to MOFs could effectively reduce the MOFs pore space [17,18].

Hence, in this paper, UiO-66-NH_2_/GO composites were in-situ synthesized with the addition of graphene oxide (GO) into UiO-66-NH_2_. Moreover, the CO_2_ adsorption performances for the UiO-66-NH_2_/GO and UiO-66-NH_2_ samples were investigated in order to discuss the influence factor effects on the structure and chemical properties, which could be beneficial for the development of high stability, high gas adsorption materials.

## 2. Experimental

### 2.1. Synthesis of UiO-66-NH_2_/GO Composites

UiO-66-NH_2_ was solvothermally synthesized based on the method of Abid et al. [19]. Zirconium chloride (ZrCl_4_, 1.47 g) and 2-aminoterephthalic acid (H_2_BDC-NH_2_, 1.06 g) were dissolved in N,N-dimethylformamide (DMF, 150 mL). Then, the mixture was transferred into a 200 mL Teflon lined stainless-steel autoclave for homogeneous reaction, which was maintained at 393 K for 24 h. The mixture was cooled to ambient temperature and repeatedly washed three times with DMF and ethanol, respectively. Yellow crystal was obtained by drying at 393 K.

The composites of UiO-66-NH_2_/GO were synthesized as the following procedure. Firstly, graphene oxide (GO) was prepared according to Hummers’ technique [17,18]. A given mass of dry scattered GO was added to 150 mL DMF solvent with ultrasonic treatment for 5 h to obtain a GO suspension. Then, ZrCl_4_ (1.47 g) and H_2_BDC-NH_2_ (1.06 g) were added to the GO suspension to synthesize UiO-66-NH_2_/GO. The expected amount of GO is 5 wt % in UiO-66-NH_2_/GO, which is the most appropriate ratio that is found in our previous studies of UiO-66/GO samples [17]. The obtained gray mixture was transferred into a 200 mL Teflon lined stainless-steel autoclave for homogeneous reaction, which was maintained at 393 K for 24 h. The mixture was cooled to ambient temperature and repeatedly washed three times with DMF and ethanol, respectively. Finally, gray crystal was obtained by drying at 393 K.

### 2.2. Characterizations

For X-ray diffraction (XRD) measurements, the samples were grounded with DMF (MOF series) or ethanol (GO series) in a small agate mortar, then, the mixture was smear-mounted onto a glass slide and was air-dried. The samples were analyzed by using a Philips X’Pert X-ray diffractometer (Cu Kα radiation, with a voltage of 40 kV and current of 40 mA, Philips (China) Investment Co. Ltd, Shanghai, China)

Infrared spectrum (FT-IR) was performed by using a MB154S-FTIR spectrometer (Bomem, Quebec City, QC, Canada) according to the attenuated total reflectance method (ATR).

Thermogravimetric analysis (TGA) was analyzed by using a SDTQ600 thermal analyzer (Perkin-Elmer Pyris Diamond, Waltham, MA, USA). Experiments were carried out under a N_2_ atmosphere (30 mL/min flow rate) at a heating rate of 10 °C/min from room temperature to 800 °C.

ASAP 2010 analyzer (Micromeritics, Norcross, GA, USA) was applied to evaluate the N_2_ adsorption isotherms at −196 °C. Before the experiment, the samples were decorated 12 h at 393 K. The surface area S_BET_, the total pore volume V_pore_, the micropore volume V_mic_, and the mesopore volume V_mes_ were obtained from the isotherms.

Scanning electron microscope (SEM) was performed on a S-4800 field emission scanning electron microscope (Hitachi High-Technologies corporation, Tokyo, Japan). Scanning was ran on a powder sample that was previously dried and also should be coated with a thinness of gold to avoid charging in the case of MOFs and the composites.

### 2.3. CO_2_ Adsorption Experiment

CO_2_ adsorption measurements under 0~1.2 bar were performed by an apparatus for physical adsorption (V-Sorbet 2008S, Beijing Jinaipu Science & Technology Co., Ltd., Beijing, China). The experiments were operated at 273 K, 298 K and 318 K. The sample tube was immersed in a heated dewar flask filled with water to control the adsorption temperature. Before the experiment, the samples (0.1–0.2 g) in the analysis chamber were subjected to a vacuum at ambient temperature for 12 h. The reversibility of the CO_2_ adsorption process on the samples was studied by multiple CO_2_ adsorption cycles. Regeneration experiments were conducted at 393 K for 12 h in vacuum, and then the corresponding adsorption isotherms were collected.

### 2.4. Chemical Stability Test

At room temperature, newly synthesized UiO-66-NH_2_/GO (0.2 g) was exposed to air humidity (70–90%) for 30 days, or 20 mL pH = 1 HCl solution (or pH = 14 in NaOH solution for 2 h. XRD testing was carried out on samples after filtration drying.

## 3. Results and Discussion

### 3.1. Structure Characterization

**Figure 1:**
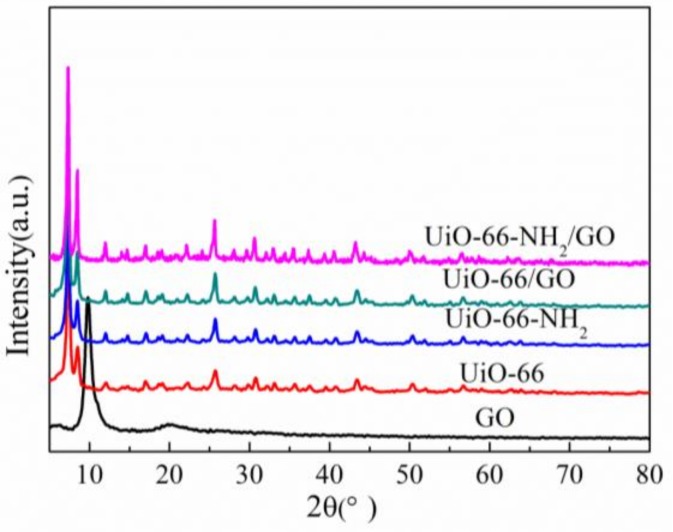
X-ray diffraction patterns of the different samples.

X-ray diffraction (XRD) of different samples is illustrated in Figure 1. As shown in Figure 1, the main diffraction peaks of UiO-66-NH_2_ are similar to those of UiO-66, revealing that the introduction of –NH_2_ groups did not change the crystal structure of matrix UiO-66, which is consistent with the results of previous reports [20,21,22]. UiO-66-NH_2_/GO composites have the same diffraction peak position and a slightly stronger peak intensity when compared with UiO-66-NH_2_. It proved that UiO-66-NH_2_ is the main component of UiO-66-NH_2_/GO composites and UiO-66-NH_2_/GO composites still maintained a good crystal structure. The main diffraction peak of GO (2θ = 10°) does not appear in UiO-66-NH_2_/GO composites, which is consistent with the situation of UiO-66/GO. The reason could be concluded that the very low content (5%) of GO in the composites, the high dispersion of GO with polar solvents (DMF), which were known to disperse GO very well, and the interaction of GO with UiO-66-NH_2_.

**Figure 2:**
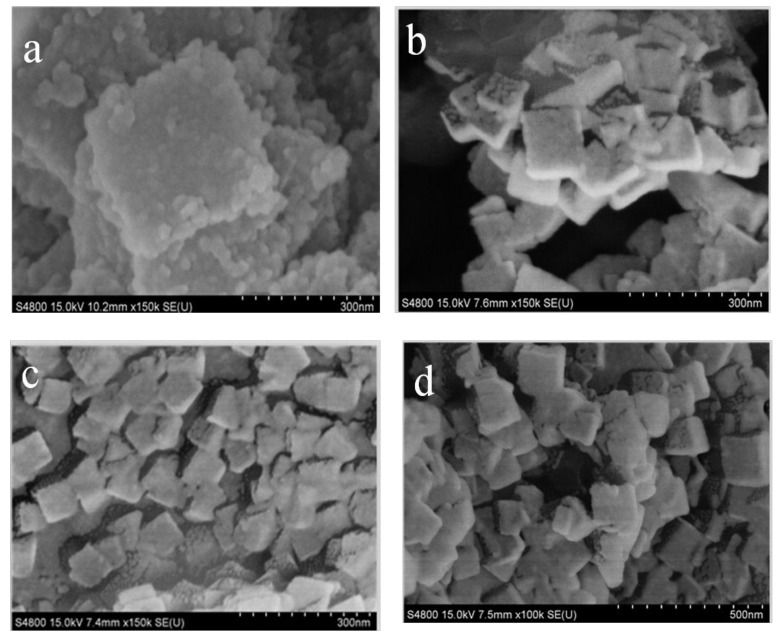
Scanning electron microscope (SEM) images of UiO-66 (**a**) [17]; UiO-66-NH_2_ (**b**); UiO-66/GO (**c**) [17]; and UiO-66-NH_2_/GO (**d**).

SEM images of different samples were showed in Figure 2. As seen from Figure 2a,b, UiO-66-NH_2_ has the same cubic structure as UiO-66 crystal. Owing to the influence of synthesis condition, the particle size of UiO-66-NH_2_ is around 150 nm, which is smaller than that of UiO-66 (300 nm). The result is in accordance with the previous reported in the literature [22]. UiO-66-NH_2_ and UiO-66/GO composites have similar cubic crystals with a much smaller particle size of approximately 80–100 nm and are of irregular appearance (as shown in Figure 2c,d), which could be attributed to the introduction of GO. It can be explained that the oxygen-containing functional groups in GO combined with Zr^4+^, inhibiting the crystal growth of UiO-66-NH_2_.

**Figure 3:**
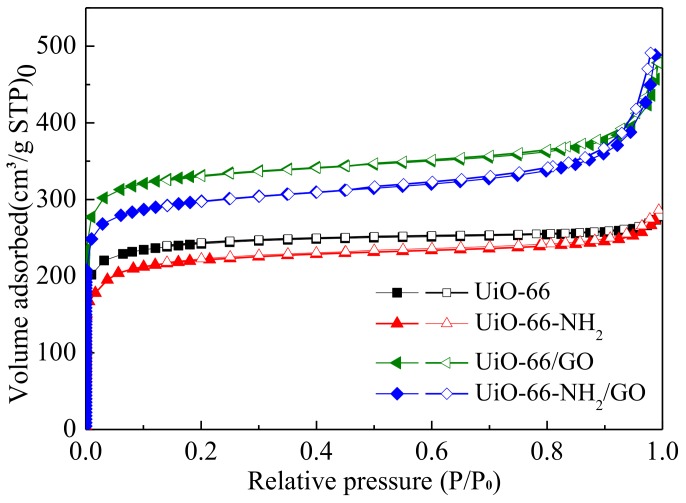
N_2_ adsorption-desorption isotherms of the different samples at 77 K.

**Figure 4:**
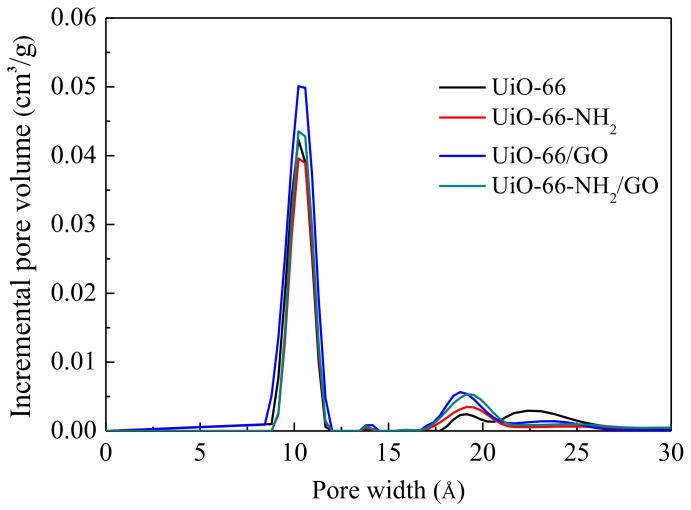
Pore size distribution of the different samples.

Figure 3 presents the N_2_ adsorption-desorption isotherm of different materials. N_2_ adsorption-desorption isothermal of all the synthetic samples reveal a typical type I isotherm. Pore structure parameters and Density Functional Theory (DFT) pore size distribution are presented in Figure 4 and Table 1.

As can be seen from Table 1, the S_BET_ and V_pore_ of UiO-66-NH_2_ is 822 m^2^/g and 0.236 cm^3^/g respectively, which is similar to that of UiO-66, in conformity with the results that were reported by Nik et al. [22], but slightly lower those that were reported by Garibay et al. [8]. This slight difference may result from the different activation temperatures. GO has no adverse effect on composite materials. Properly adding GO significantly improves the S_BET_ and V_por_ of UiO-66/GO. For S_BET_, V_pore_*,* and V_mic_ of UiO-66-NH_2_/GO are 1052 m^2^/g, 0.345 cm^3^/g, and 0.286 cm^3^/g, respectively, which increases by 28%, 46%, and 46% than UiO-66-NH_2_. The incorporation of GO does not negatively affect the porosity of UiO-66-NH_2_, it is in accordance with the previous report. The new pores could generate on the interface between the GO layers and the MOF “blocks”, which could improve the surface area.

As shown in Figure 4, the pore size of the composite material is 8~12 Å. In this range, the pore size distribution of UiO-66/GO and UiO-66-NH_2_/GO is more than the corresponding parent material UiO-66 and UiO-66-NH_2_, which is good for CO_2_ adsorption. It can also be seen that the pore sizes of these materials are partly distributed between 16 Å and 22 Å. During this interval, the pore size distribution of UiO-66-NH_2_ and UiO-66 is similar, and that of UiO-66/GO and UiO-66-NH_2_/GO is similar. The pore volume of the composite materials is higher than that of the parent material, which is also good for CO_2_ adsorption.

### 3.2. Chemical Characteristics

**Figure 5:**
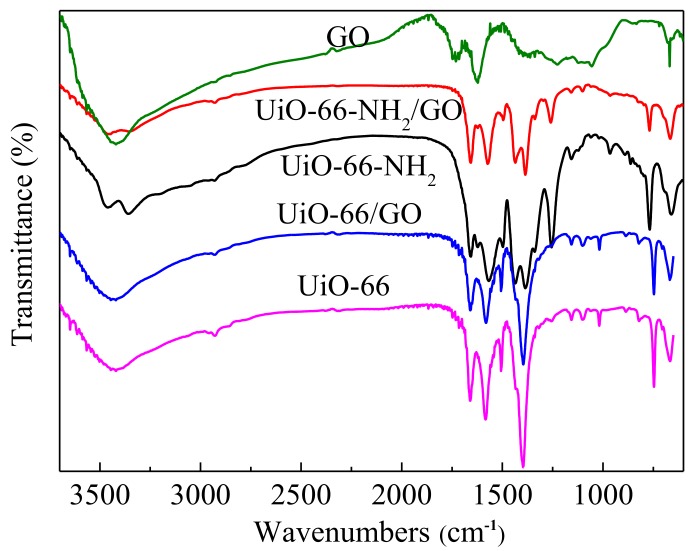
FT-IR spectra of the different samples.

Figure 5 is the FT-IR spectra of different samples. In the spectrum of UiO-66-NH_2_, new peaks appeared at 3461 cm^−1^ and 3361 cm^−1^, respectively, when compared with UiO-66, corresponding to the symmetric and asymmetric vibration peak of –NH_2_ [23]. The peak at 1621 cm^−1^ corresponds to the N–H bending vibration. The peaks at 1482 cm^−1^ and 1382 cm^−1^ can be attributed to the N–H bending vibration and C–N stretching vibration, respectively [12]. The IR spectrum of UiO-66/GO was almost the same as that of UiO-66, with a slight decrease in strength.

When comparing to the UiO-66-NH_2_, the spectrum of UiO-66-NH_2_/GO composites was relatively unchanged, while the peak strength decreased slightly. The main peaks of GO also do not appear in the composite materials. The oxygen functional groups on the GO layers could bond with the open metal sites of UiO-66-NH_2_, resulting in the band disappearance of GO over UiO-66-NH_2_/GO composites in the FTIR spectra. In addition, small changes in the composite materials were observed at 1158 cm^−1^, 893 cm^−1^, 961 cm^−1^, and 698 cm^−1^. The reason for this is that the introduction of functional groups that are carried by the GO surface and carboxyl coordinated with metal ion slightly changed the chemical environment of the Terephthalic acid ligand.

### 3.3. Stability Analysis

**Figure 6:**
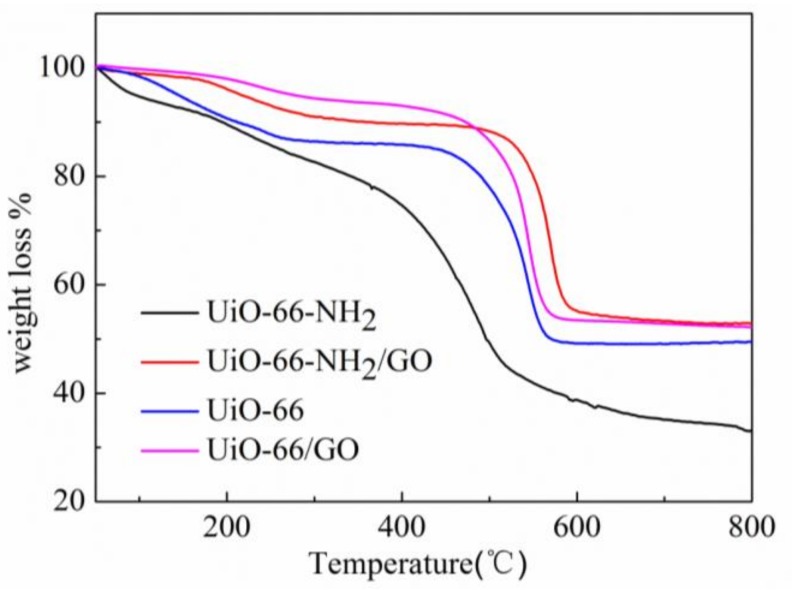
Thermo-gravimetric analysis for the different samples.

Figure 6 shows the TG curves of the different samples. The thermal stability of UiO-66-NH_2_ decreased when compared with that of UiO-66. The terephthalic acid ligand in UiO-66 skeleton materials decomposed above 480 °C, but the decomposition of amino terephthalic acid ligand occurred from 380 °C, which is 100 °C lower than that of UiO-66 materials. This is consistent with the UiO-66-NH_2_ collapse of skeleton temperature, as reported by the literature [8]. The decomposition temperature of other types of amine-MOF is also lower than that of the matrix [24,25]. Finally, when the temperature reaches 650 °C, the UiO-66-NH_2_ has the largest quality loss of approximately 65 wt %, and the quality loss of the UiO-66 is about 52 wt %. This shows that the thermostability of the latter is better. In addition, the thermostability of UiO-66-NH_2_/GO composites is better than UiO-66, which is similar to UiO-66/GO composites. Their decomposition temperature is in the range of 500–650 °C, with a similar quality loss of 47 wt %. To sum up, the thermal stability of UiO-66-NH_2_/GO composites is obviously higher than that of UiO-66-NH_2_. The addition of the right amount of GO clearly improved the thermostability of UiO-66-NH_2_.

**Figure 7:**
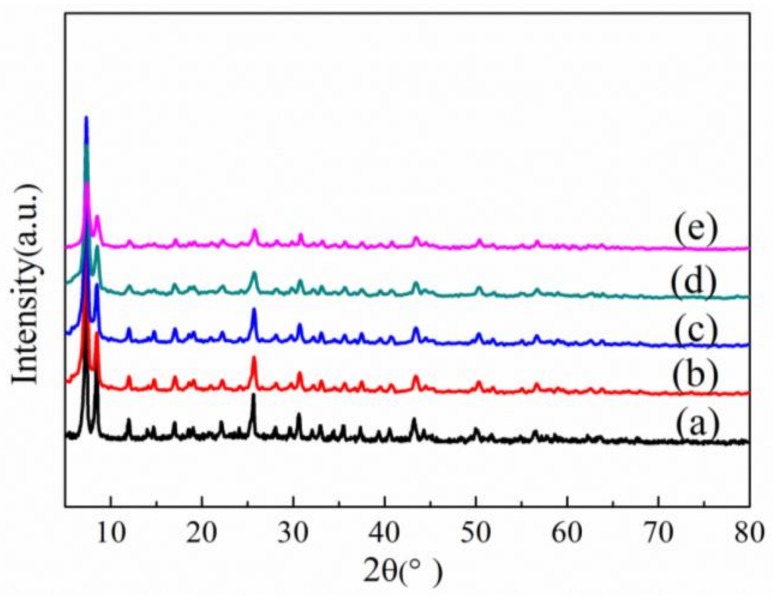
XRD patterns for UiO-66-NH_2_/GO: (**a**) as-prepared; (**b**) desolvated; (**c**) in air for 30 days; (**d**) soaked in water for 10 days; (**e**) immersed in in HCl solution (pH = 1) for 2 h; and, (**f**) NaOH solution (pH = 14) for 2 h.

In addition to the above investigation of the thermal stability, we also studied the chemical stability of the UiO-66-NH_2_/GO. The stability of UiO-66-NH_2_/GO in the air, water, acid, and alkali qualitative is characterized by XRD. As can be seen from Figure 7b,c, after the UiO-66-NH_2_/GO exposed in the air for 30 days, or is soaked in water for 10 days at room temperature, the crystal structure did not change, which proved that the UiO-66-NH_2_/GO has a high water stability that is the same as that of matrix UiO-66 and UiO-66/GO [17]. The introduction of GO means that the UiO-66-NH_2_ can improve the stability of the network structure under the condition of humidity.

As shown in Figure 7d,e, after the UiO-66-NH_2_/GO was soaked in HCl solution (pH = 1) and NaOH solution (pH = 14) for 2 h, the strength of the main peak got slightly smaller, but was still able to maintain crystal structure. According to the literature, after soaking in the same condition of the NaOH solution, the skeleton of UiO-66-NH_2_, UiO-66-NO_2_, and UiO-66-Br completely collapsed, whereas the UiO-66 structure was damaged, and it was converted into a state of lower crystallinity [11]. A chemical stability test showed that UiO-66-NH_2_/GO have excellent water resistance, acid resistance, and alkali resistance. Among them, the alkali resistance has a significant increase when compared with the other reported UiO-66 type of MOFs.

### 3.4. Low Pressure CO_2_ Adsorption

**Figure 8:**
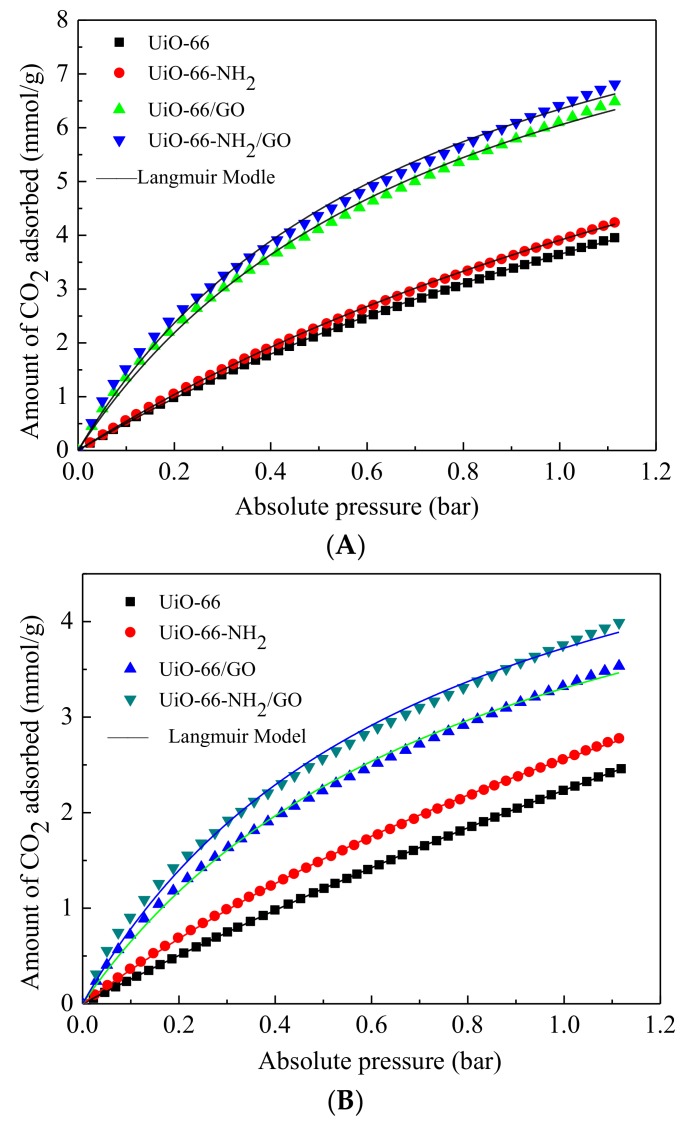

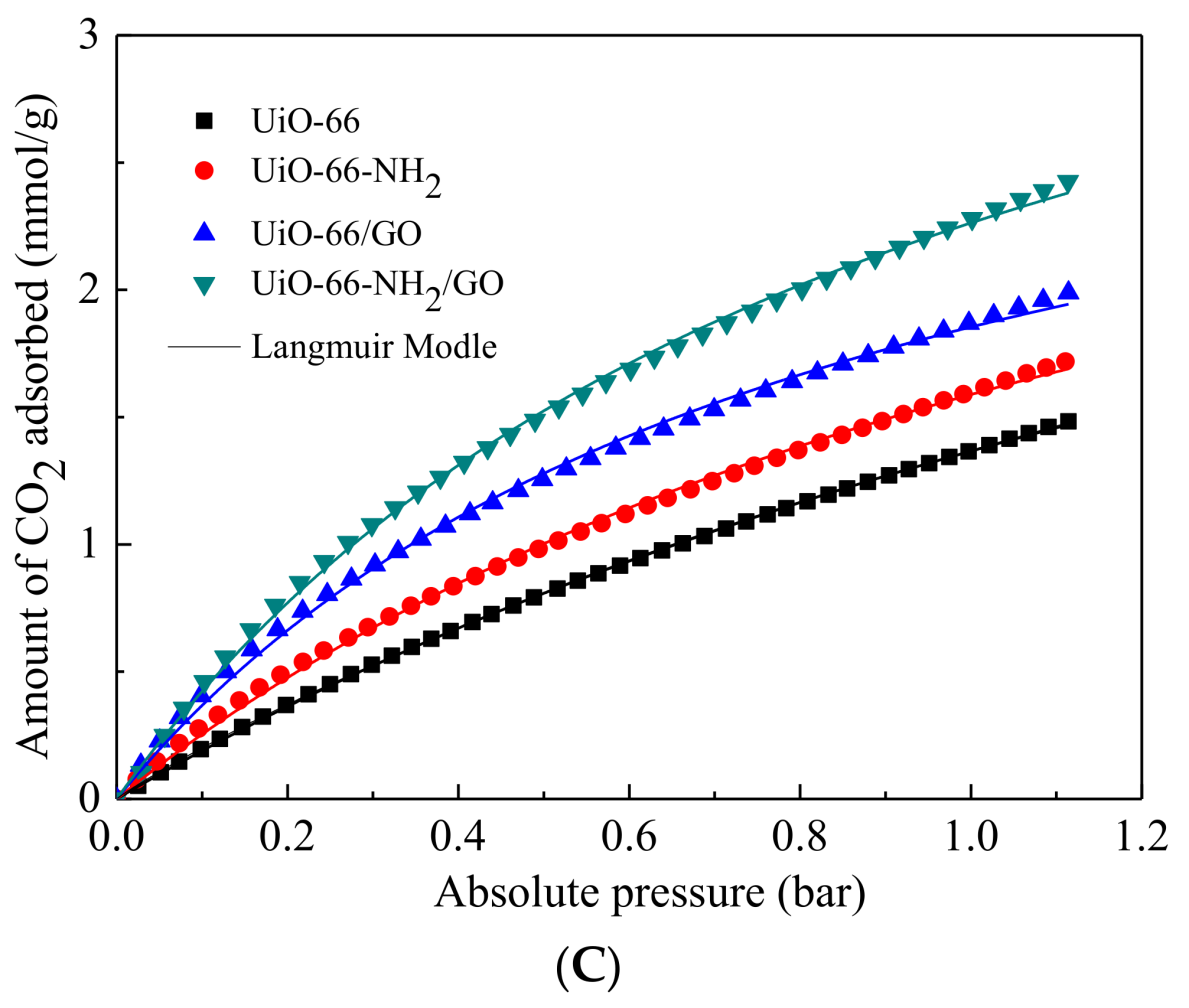
CO_2_ adsorption isotherms measured at 273 K (**A**); 298 K (**B**); and, 318 K (**C**).

The CO_2_ adsorption performance of the samples was elevated using a physical adsorption instrument under the condition of low voltage static. Before the gas adsorption test, all of the samples were dried under vacuum for 12 h at 393 K to completely remove the solvent molecules from inorganic metal element Zr [13]. CO_2_ adsorption isotherms of different samples are shown in Figure 8. The tests were carried out at 273 K, 298 K, and 318 K, respectively, and 0~1.2 bar.

The CO_2_ adsorption capacities of all of the samples decrease with the increase of temperature. The CO_2_ uptakes of all the samples under three temperatures followed the same order: UiO-66-NH_2_/GO > UiO-66/GO > UiO-66-NH_2_ > UiO-66. As can be seen from the isotherms, the CO_2_ adsorption capacity of UiO-66 and UiO-66/GO clearly increased with the decrease of temperature, which proved that the adsorption mechanism can be assigned to physical adsorption. The incorporation of GO to UiO-66 led to the increase of the mesopore volume and micropore volume. The mesopore was in favor of the transmission of CO_2_ to the adsorption site with high energy and the micropore was in favor of the strong retention of CO_2_ molecules. As showed in Figure 8, the adsorption capacity of UiO-66-NH_2_ at 273 K below 1.0 bar is 3.93 mmol/g, which is 7.3% higher than that of UiO-66 (3.66 mmol/g). A similar situation occurs when the adsorption temperature increases to 318 K. The UiO-66-NH_2_ adsorption capacity is 1.60 mmol/g, which is 16.0% higher than that of UiO-66 1.38 mmol/g. The UiO-66-NH_2_/GO adsorption capacity at 273 K is 6.41 mmol/g, which is 5.1% higher than that of UiO-66/GO (6.10 mmol/g), while the UiO-66-NH_2_/GO adsorption capacity at 318 K is 2.26 mmol/g, which is 20.2% higher than that of UiO-66 1.88 mmol/g. As known in Table 1, the S_BET_ of UiO-66-NH_2_ is a bit smaller than that of UiO-66. Similarly, the specific surface area of UiO-66-NH_2_/GO is slightly smaller than that of UiO-66/GO. Moreover, in terms of CO_2_ adsorption capacity, the opposite is true: UiO-66-NH_2_ is above UiO-66, and UiO-66-NH_2_/GO is higher than UiO-66/GO. This phenomenon shows that, for UiO-66-NH_2_ and UiO-66-NH_2_/GO, in addition to the physical adsorption sites of CO_2_ adsorption, there are other adsorption sites, and the adsorption reaction between CO_2_ and amino groups improves the CO_2_ adsorption capacity. Moreover, the high temperature is advantageous in terms of the reaction on the adsorption sites and being conducive to the physical adsorption at low temperature, high temperature reaction adsorption sites when strength is more obvious. The existence of reaction adsorption sites results in strong interaction and better adsorption selectivity between the adsorbate and adsorbent, which favors the important factors of gas purification.

In Table 2, several traditional adsorbent and synthetic samples for CO_2_ adsorption capacity under 1.0 bar are listed. As can be seen from the Table 2, when compared with other typical porous materials, such as 13 x molecular sieve, activated carbon adsorption, and some common MOFs, the UiO-66-NH_2_/GO composite has a higher CO_2_ adsorption capacity. Therefore, the UiO-66-NH_2_/GO composite has certain industrial application prospects.

In order to determine the reason for the difference in CO_2_ adsorption capacity, the calculation of CO_2_ adsorption heat was carried out. When compared with UiO-66, UiO-66-NH_2_, and UiO-66/GO, the CO_2_ adsorption capacity of UiO-66-NH_2_/GO has an excellent performance. The reason for this may be related to the adsorption heat Δ*H*.

Langmuir isotherm equation had been proposed to describe the experimental data, according to the following Langmuir equation:

(1) $$Q_{e}{=Q}_{m}\frac{{\mathrm{bP}}_{e}}{{1+\mathrm{bP}}_{e}}$$

where Q_e_ and P_e_ are equilibrium adsorption and pressure, respectively. Q_m_ is the monolayer capacity and b is the Langmuir equilibrium constant. Isotherm parameters at all of the temperatures are summarized in Table 3.

**Figure 9:**
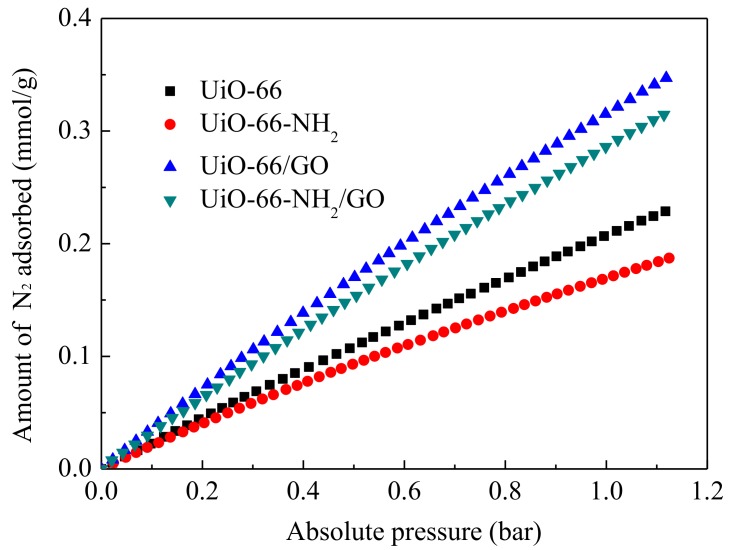
N_2_ adsorption isotherms measured at 298 K on the sample.

From the isothermal measurements performed at three different temperatures (Figure 8 and Figure 9), it is possible to calculate the net isosteric heat of adsorption from Clausius-Clapeyron equation [30]:

lnP = −(ΔH/RT) + C(2)

where ΔH is the amount of adsorption heat (kJ/mol), p is the pressure (MPa), *T* is the temperature (K), *R* is the gas constant, and *C* is integration constant. The lnP plot of 1/T is a straight line with slope—(ΔH/R). According to the low voltage (0.01 MPa to 0.1 MPa), under the 273 K, 298 K and 318 K adsorption isotherm, we obtain relevant data generation according to the type, and CO_2_ isothermal adsorption heat is calculated. The isosteric heat of adsorptions for CO_2_ were 24.3~22.9 kJ/mol for UiO-66, 24.6~23.2 kJ/mol for UiO-66-NH_2_, 28.9~27.8 kJ/mol for UiO-66/GO, and 30.5~29.4 kJ/mol for UiO-66-NH_2_/GO, depending on the degree of CO_2_ loading. There was no significant difference in Q values and the maximum variation between the highest and lowest values did not exceed 5%. Therefore, an average *Q* was calculated. The average isosteric heat of adsorption for CO_2_ on the samples studied. UiO-66, UiO-66-NH_2_, UiO-66/GO, and UiO-66-NH_2_/GO CO_2_ isothermal adsorption heat is, respectively, 23.5 kJ/mol, 24.2 kJ/mol, 28.3 kJ/mol, and 29.9 kJ/mol. Among them, the UiO-66 and UiO-66-NH_2_ CO_2_ isothermal adsorption heat is the same as the literature. Hui et al. [31] measured CO_2_ UiO-66 when the high coverage adsorption heat is 22 kJ/mol. Aprea et al. [32] reported that the 13x molecular sieve CO_2_ isothermal adsorption heat is 32.5 kJ/mol. Generally, the smaller the adsorption heat of the materials, the lower renewable energy. However, for low adsorption heat, the adsorption selectivity will decrease, leading to the capture of the lower gas purity. The UiO-66-NH_2_/GO CO_2_ isothermal adsorption heat is 29.9 kJ/mol, which is higher than the UiO-66-NH_2_ CO_2_ isothermal adsorption heat of 24.2 kJ/mol. The UiO-66/GO CO_2_ isothermal adsorption heat is 28.3 kJ/mol, which is higher than that of UiO-66 isothermal adsorption heat 23.5 kJ/mol. This is mainly due to holes or defects in the interface between the MOF and the GO in the crystal structure, boosting the interaction between the skeleton and CO_2_, and thus increasing the CO_2_ adsorption capacity. At the same time, CO_2_ intermolecular interactions in the channel may also increase △H. The UiO-66-NH_2_ CO_2_ isothermal adsorption heat is 24.2 kJ/mol, which is slightly higher than that of the UiO-66 CO_2_ isothermal adsorption heat of 23.5 kJ/mol. The UiO-66-NH_2_/GO CO_2_ isothermal adsorption heat is 29.9 kJ/mol, which is slightly higher than the UiO-66/GO isothermal adsorption heat of 28.3 kJ/mol. This can be attributed to the existence of -NH_2_ and the reaction adsorption sites between CO_2_, raising the heat of adsorption. The interaction between CO_2_ is enhanced, the CO_2_ adsorption capacity increases, and thus, eventually, there was a corresponding conformity. 

### 3.5. Adsorption Selectivity of CO_2_/N_2_

UiO-66-NH_2_/GO may be a potential adsorbent for flue gas containing CO_2_/N_2_ in view of its high stability and CO_2_ adsorption capacity. In order to get N_2_ adsorption isotherms, the sample is tested at 298 K in the pressure range from 0 to 1 bar. As shown in Figure 9, the adsorption capacity order of N_2_ on the sample is: UiO-66/GO > UiO-66-NH_2_/GO > UiO-66 > UiO-66-NH_2_. This is different from that of CO_2_ on the sample, the latter is: UiO-66-NH_2_/GO > UiO-66/GO > UiO-66-NH_2_ > UiO-66. When compared with parent materials, the N_2_ adsorption capacity of UiO-66-NH_2_ and UiO-66-NH_2_/GO composites decreases, which is helpful for improving the CO_2_/N_2_ adsorption selectivity of the sample.

**Figure 10:**
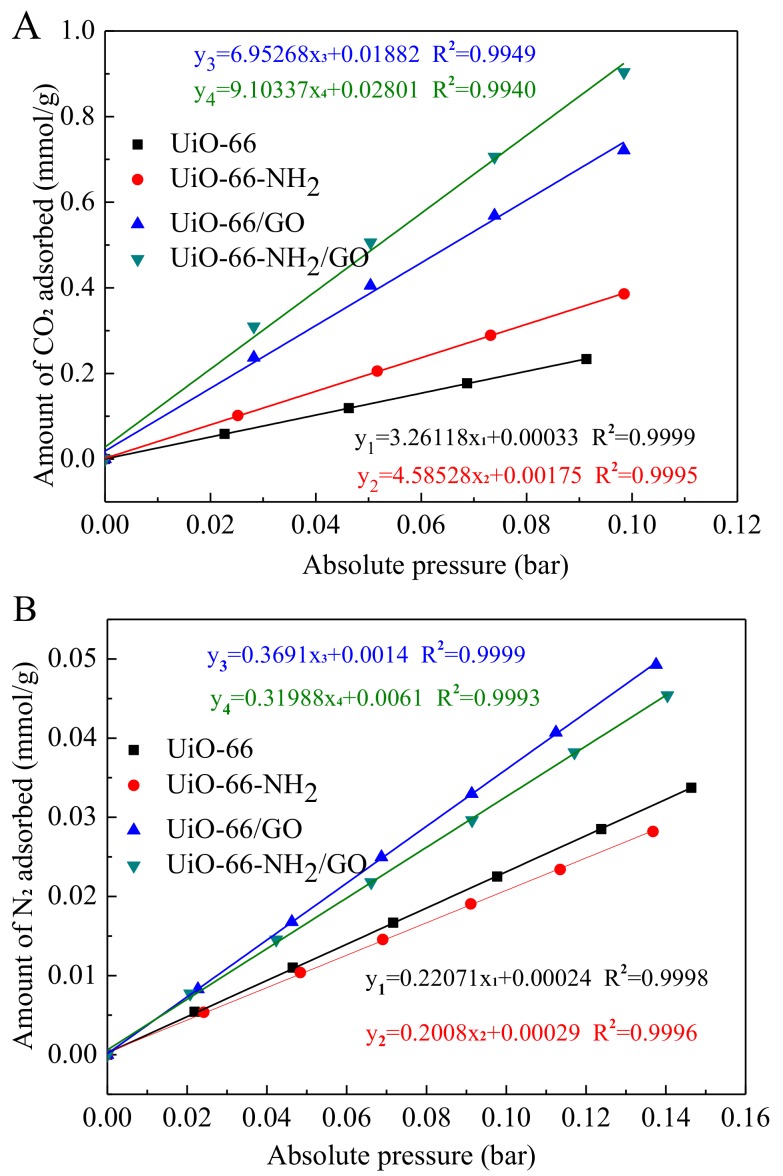
Initial slopes calculation for CO_2_ (**A**) and N_2_ (**B**) collected at 298 K.

According to the CO_2_ and N_2_ adsorption isotherm of the samples in Figure 8B and Figure 9, CO_2_/N_2_ selectivity can be calculated. The initial slope (Henry’s constant) of the CO_2_ and N_2_ adsorption isotherm at the low pressure section can be used to calculate the selectivity factor and the single-component adsorption isotherm can be used to estimate the selectivity factor. The selectivity factor is defined as the ratio of the initial slope of CO_2_ and N_2_ [33]. Figure 10 shows the result according to the initial slope of CO_2_ and N_2_ adsorption isotherm.

The selectivities of UiO-66, UiO-66/GO, UiO-66-NH_2_, and UiO-66-NH_2_/GO to CO_2_/N_2_ were 14.78, 18.84, 22.83, and 28.45. The selectivity of UiO-66 to CO_2_/N_2_ is 14.78. The selectivity of UiO-66-NH_2_ to CO_2_/N_2_ is 22.83, which has increased by 54.5%. The selectivity of UiO-66/GO to CO_2_/N_2_ is 18.84 and UiO-66-NH_2_/GO to CO_2_/N_2_ is 28.45. The latter has increased by 51.0%. Similar to the MOFs of the amine ligand, the selectivity of both UiO-66-NH_2_ and UiO-66-NH_2_/GO composites clearly improve. This is because the adsorption of UiO-66-NH_2_ and UiO-66-NH_2_/GO to CO_2_ contain chemical reaction adsorption sites, in addition to physical adsorption sites because of -NH_2_, which makes their adsorption capacity to CO_2_ increase significantly. Furthermore, the adsorption capacity of UiO-66-NH_2_ and UiO-66-NH_2_/GO to N_2_ decrease. The selectivity order of the sample to CO_2_/N_2_ is: UiO-66-NH_2_/GO > UiO-66-NH_2_ > UiO-66/GO > UiO-66. Saha et al. [34] reported that the selectivity of MOF-5 and MOF-177 to CO_2_/N_2_ is 17.48 and 17.73, respectively, at 298 K and 1 bar. When compared with MOF-5 and MOF-177, the selectivity of UiO-66-NH_2_ and UiO-66-NH_2_/GO synthesized to CO_2_/N_2_ is much higher. To sum up, UiO-66-NH_2_/GO and UiO-66-NH_2_ have good prospects for industrial applications.

### 3.6. Multiple Cycles of CO_2_ Adsorption–Desorption

**Figure 11:**
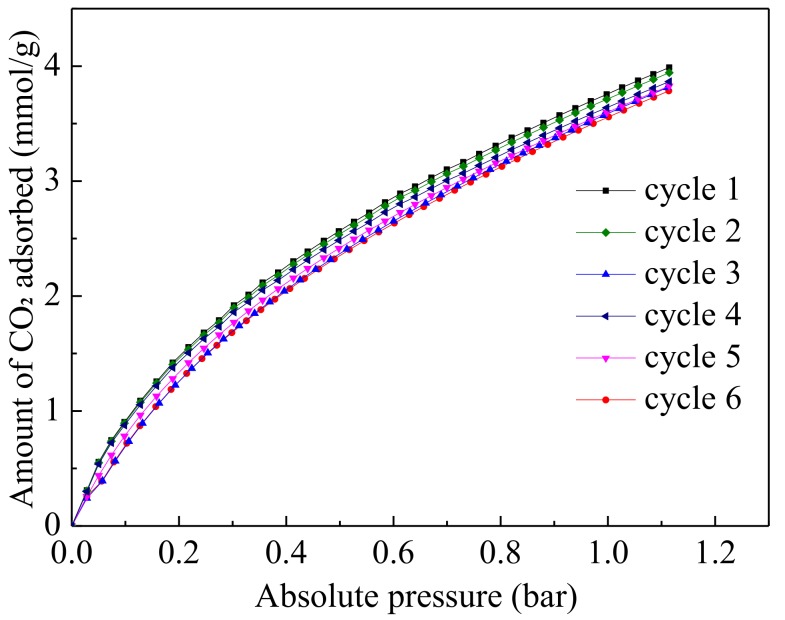
Six cycles of CO_2_ adsorption isotherms on UiO-66-NH_2_/GO at 298 K.

Figure 11 depicts the reversibility of the CO_2_ adsorption process on UiO-66-NH_2_/GO at 298 K from 0 bar to 1.2 bar. The isotherms in the diagram have been obtained after 12 h heating at 393 K in the vacuum condition. The adsorption capacity of the UiO-66-NH_2_/GO was lowered by only 4% after six cycles, from 3.80 to 3.64 mmol/g at 298 K and 1.0 bar. The six isotherms overlapped partly. The cyclical data reveals that the new UiO-66-NH_2_/GO material that is synthesized in this work is a more stable adsorbent after six adsorption/desorption cycles under mild regeneration conditions.

## 4. Conclusions

In this work, new composite materials of graphene oxide (GO)-incorporated metal-organic framework (MOF)(UiO-66-NH_2_/GO) were in-situ synthesized, and exhibited enhanced high performances for CO_2_ capture. The sample stabilities of this new sample were also investigated in the presence of water attack or acid treatment or base treatment. In terms of stability, the results were good. UiO-66-NH_2_/GO have been characterized by kinds of technical indicators and have been tested for CO_2_ adsorption, with comparison of counterparts UiO-66-NH_2_, UiO-66/GO, and UiO-66 samples. S_BET_, V_pore_, and V_mic_ of UiO-66-NH_2_/GO are, respectively, 1052 m^2^/g, 0.345 cm^3^/g, and 0.286 cm^3^/g larger than UiO-66-NH_2_ but slightly smaller than UiO-66/GO. The CO_2_ absorbance of UiO-66-NH_2_/GO achieves 6.41 mmol/g at 273 K, which is 5.1% higher than that of UiO-66/GO (6.10 mmol/g). In addition to physical adsorption sites, chemical adsorption sites (amino groups) also improved the CO_2_ adsorption capacity. CO_2_ adsorption heat and CO_2_/N_2_ selectivity was 29.9 kJ/mol and 28.45, which is higher than the selectivity of UiO-66-NH_2_ and UiO-66/GO. UiO-66-NH_2_/GO exhibited better optimized CO_2_ adsorption and CO_2_/N_2_ selectivity. So, UiO-66-NH_2_/GO composites has a potential application in CO_2_ capture technologies to alleviate the increase in temperature of the earth’s atmosphere.

## Figures and Tables

**Table 1:** Data of porous structure of the different samples.

Sample	S_BET_ (m^2^/g)	V_pore_ (cm^3^/g)	V_mic_ (cm^3^/g)	V_mic_/V_pore_ (%)
UiO-66	838	0.245	0.224	91
UiO-66-NH_2_	822	0.236	0.214	90
UiO-66/GO	1184	0.384	0.304	79
UiO-66-NH_2_/GO	1052	0.345	0.286	83

S_BET_ is the BET surface area; V_pore_ is total pore volume; V_mic_ is micropore volume.

**Table 2:** Adsorption capacity of the prepared composite and some other adsorbents for selected CO_2_ reported from the literature at 1 bar.

Sample	Chemical Formula	Q (mmol/g)	Temperature (K)	Ref.
MOF-5	Zn_4_O(BDC)_3_	2.10	296	[26]
IRMOF-1	Zn_4_O(BDC)_3_	1.92	208	[2]
MOF-177	Zn_4_O(BTB)_2_	0.8	298	[27]
zeolite 13X	-	1.77	293	[28]
Activated carbon	-	1.5	298	[29]
UiO-66	Zr_6_O_4_(OH)(BDC)_6_	1.77	298	[4]
UiO-66-NH_2_	Zr_6_O_4_(OH)(BDC-NH_2_)_6_	3.05	298	[4]
UiO-66	Zr_6_O_4_(OH)(BDC)_6_	2.27	298	present work
UiO-66/GO	-	3.37	298	present work
UiO-66-NH_2_	Zr_6_O_4_(OH)(BDC-NH_2_)_6_	2.59	298	present work
UiO-66-NH_2_/GO	-	3.80	298	present work

Q is CO_2_ adsorption capacities.

**Table 3:** Data of Langmuir isotherm for CO_2_ adsorption on the different samples.

Sample	273 K			298 K			318 K		
	Q_m_	b	R^2^	Q_m_	b	R^2^	Q_m_	b	R^2^
UiO-66	11.78	0.45	0.999	15.49	0.17	0.999	4.42	0.45	0.999
UiO-66-NH_2_	12.63	0.45	0.999	8.28	0.45	0.999	3.82	0.71	0.998
UiO-66/GO	10.83	1.26	0.997	6.03	1.20	0.997	3.37	1.22	0.997
UiO-66-NH_2_/GO	10.92	1.39	0.996	6.38	1.41	0.996	4.39	1.06	0.998
